# Off-pump coronary artery bypass with heparin in a patient with a history of heparin-induced thrombocytopenia: a case report

**DOI:** 10.1186/s40792-021-01339-9

**Published:** 2021-12-20

**Authors:** Yuya Ito, Aya Saito, Yuki Shirai, Noboru Motomura

**Affiliations:** Department of Cardiovascular Surgery, Toho University Sakura Medical Center, 564-1 Shimoshizu, Sakura, Chiba 285-8741 Japan

**Keywords:** Coronary Artery Bypass Grafting, Heparin, Thrombocytopenia, Argatroban, Angina Pectoris

## Abstract

**Background:**

Cardiovascular surgery for patients with a history of heparin-induced thrombocytopenia (HIT) with thrombosis requires careful perioperative anticoagulation therapy. When cardiovascular surgery is required for patients having ‘remote’ HIT, such as those who had a history of HIT and platelet factor-4/heparin antibodies turned out to be negative, it is recommended that re-exposure to heparin should be limited only to the intraoperative phase. However, few case reports have described detailed strategies for perioperative anticoagulation regimens.

**Case presentation:**

We present the case of a 76-year-old woman, presenting with unstable angina pectoris and requiring coronary artery bypass grafting. She had a history of cardiac resuscitation and percutaneous coronary intervention for unstable angina pectoris with ventricular tachycardia 7 years prior, which caused HIT with thrombosis resulting in amputation of four fingers. On admission, platelet factor-4/heparin antibodies, biomarkers for HIT were not detected; the platelet count was 18.0 × 10^4^/µl. Off-pump coronary artery bypass grafting was performed using heparin; argatroban infusion was continued until 9 h prior to the operation and restarted 3 h postoperatively, bridged with regular warfarin from 4 days to 3 months postoperatively. Platelet factor-4 /heparin antibodies were detected on postoperative day 8 without any clinical symptoms and became negative by day 91.

**Conclusion:**

We consider this anticoagulation strategy is effective especially in countries, where bivalirudin is not available. Re-exposure to heparin in cardiovascular surgery for patients with a history of ‘remote HIT’ is reasonable, and appropriate anticoagulation is important for an uneventful postoperative course.

## Background

Heparin-induced thrombocytopenia (HIT) is an antibody-mediated adverse effect of heparin, causing devastating thromboembolic events which result in adverse outcomes [1, 2]. One of the characteristics of the HIT immune response is its transient nature, with a median time to negative of 85 days [3].

The incidence of HIT in cardiac surgery is 1–2.4% [1–3], with venous thrombosis being the most common complication; in addition, 17–55% of untreated patients with thrombocytopenia develop deep vein thrombosis (DVT) and/or pulmonary embolism (PE) [1]. Arterial thrombotic events, including limb embolism, stroke, and myocardial infarction (MI), also occur, but less often (3–10%) [1]. The mortality rate of patients with HIT with thrombosis (HIT-T) is 5–10%, which usually occurs as a result of thrombotic complications [4, 5].

As per the recommendations of the American College of Chest Physicians (ACCP), non-heparin anticoagulants, such as bivalirudin and argatroban, are the first-line anticoagulation therapy in patients with a history of HIT [1]. The EVOLUTION-On study reported no difference in the clinical outcome, which was defined as the absence of death, Q-wave MI, stroke, or repeat coronary revascularization, between heparin and bivalirudin in cardiopulmonary bypass (CPB) [9]. Unfortunately, bivalirudin is not available in Japan; therefore, argatroban is the first choice. When performing cardiac surgery in a history of HIT, distinguishing the phase of HIT (acute, subacute, remote) is important. When a patient has had prior development of platelet factor-4/heparin antibodies (PF4/H Ab) and has subsequently tested negative, they are termed as having ‘‘remote’’ HIT [1, 6]. Moreover, when cardiovascular surgery is required for patients having ‘‘remote’’ HIT, it is recommended that re-exposure to heparin should be limited only to the intraoperative phase. However, few case reports have described detailed strategies for perioperative anticoagulation regimens, especially in countries, where anticoagulants other than heparin, such as bivalirudin, are not available.

Herein, we present a case of off-pump coronary artery bypass (OPCAB) with a combination of heparin and perioperative argatroban use in a patient with a history of HIT-T.

## Case presentation

A 76-year-old woman with a history of HIT-T was admitted to our department for unstable angina pectoris (UAP); coronary artery bypass grafting (CABG) was indicated. Her first admission by the cardiologist was 7 years prior for UAP with congestive heart failure, and diagnosed with three-vessel coronary artery disease. She was mechanically ventilated; intra-aortic balloon pumping (IABP) and percutaneous coronary intervention (PCI) with a bare-metal stent to the right coronary artery (RCA) were performed. Post-intervention, continuous hemodiafiltration (CHDF) with heparin was continued. Repeated heparin use caused gangrene and amputation of four fingers; she was subsequently diagnosed with HIT-T, and a temporary tracheostomy was required. Moreover, due to her poor general condition, PCI for residual coronary disease was not performed.

Other comorbidities included insulin-dependent diabetes mellitus (hemoglobin A1c = 6.4%), chronic kidney disease (eGFR = 15 ml/min/1.73 m^2^), pulmonary hypertension, and peripheral artery disease (left carotid artery and left radial artery stenosis). There was no family history of coronary disease, allergy, or history of smoking.

Her left ventricular function was moderately impaired (left ventricular ejection fraction, 44%). The preoperative platelet count was 18.0 × 10^4^/μl, and platelet factor PF4/H Ab was negative; thus, she was diagnosed with ‘‘remote’’ HIT [1, 6]. The estimated postoperative mortality by Japan SCORE II (which does not include entry for the thromboembolism) was 4.9%, and the estimated risk of mortality and morbidity was 28%.

On admission, aspirin and clopidogrel were substituted for argatroban, and argatroban was used during coronary angiography. Target activated partial thromboplastin time (aPTT) was twice the baseline; thus, argatroban was administered until 9 h prior to the operation. Surgery was performed under general anesthesia, and a median sternotomy approach was used. Simultaneously, the right femoral artery and vein were secured by introducing peripheral mechanical circulatory support, avoiding central cannulation for cardiopulmonary bypass establishment.

**Fig. 1:**
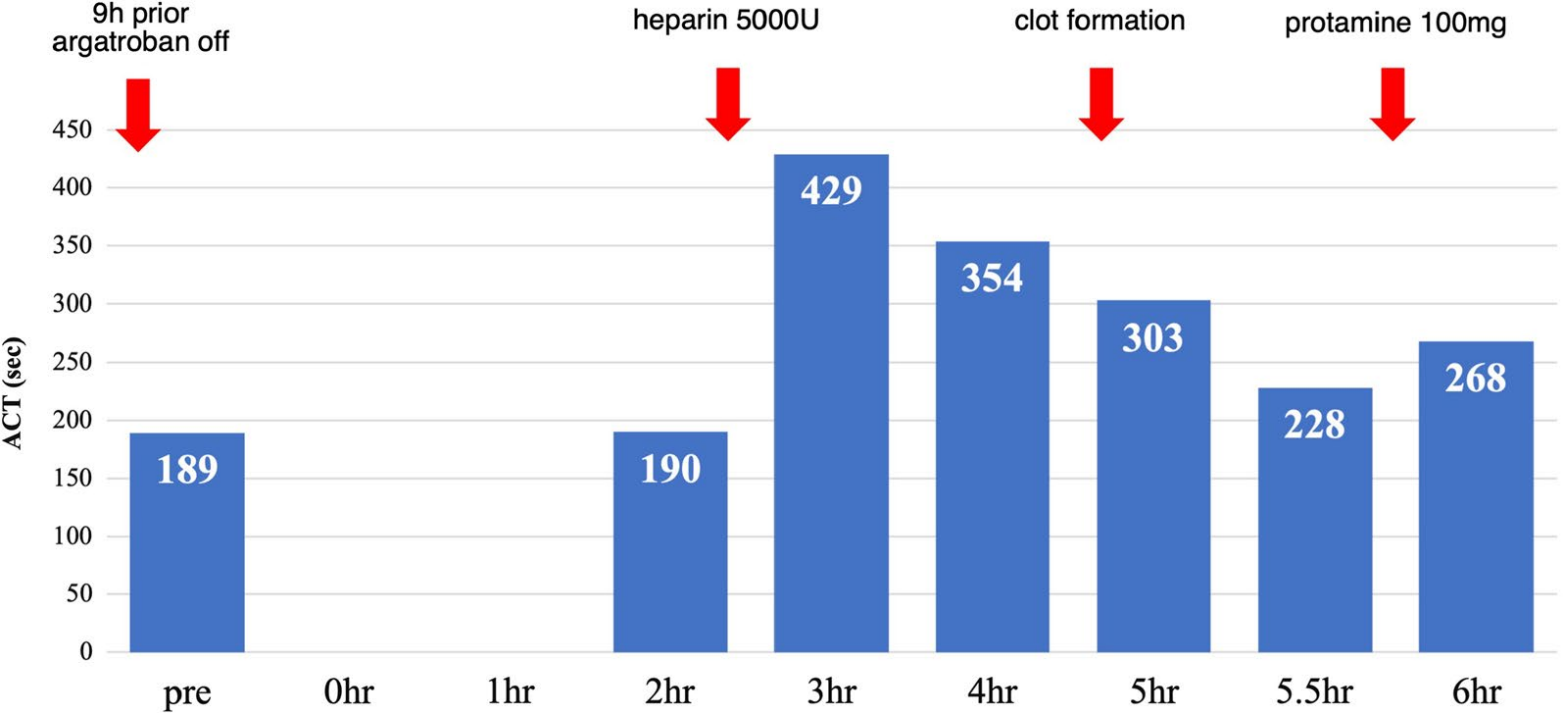
Course of ACT and anticoagulation**.** Course of ACT and anticoagulation. Dual antiplatelet therapy was converted to argatroban preoperatively. The dose of heparin and the reaction of ACT were as usual. Clot formation was observed in the surgical field when the ACT was around 300 s. Even when sufficient protamine was used, the ACT was prolonged. ACT, activated clotting time

A bolus of 5000-unit unfractionated heparin was administered when anastomosis was started, and activated clotting time (ACT) was maintained between 189 s (control score) and 429 s (Fig. 1). After all coronary anastomoses (RITA-LAD, LITA-LPL, SVG-4PD) were completed, 100 mg protamine was administered; however, the ACT did not normalize fully (Fig. 1). The surgery was uneventful, and the patient was extubated in the operating theater. The intra and postoperative blood losses were 495 cc and 358 cc/16 h, respectively.

**Fig. 2:**
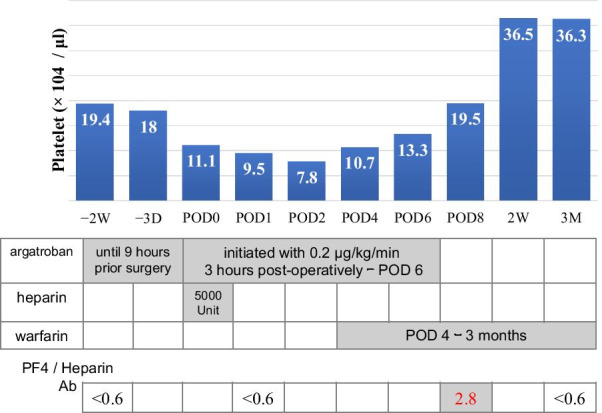
Course of platelet count, anticoagulation therapy, and PF4/H antibody**.** Platelet count reduced to 7.8 × 10^4^/μl on POD 2 and recovered gradually. Argatroban was initiated 3 h postoperatively and continued until POD 6. Warfarin was also started when platelet count exceeded 10 × 10^4^/μl on POD 4 and continued for 3 months. PF4/H Ab turned positive on POD 8, but disappeared 91 days later. POD, postoperative day; PF4/H Ab, platelet factor-4/heparin antibodies

Furthermore, the postoperative anticoagulation regimen was based on the ACCP guideline [1]. Argatroban was initiated at 0.2µg/kg/min (this value is in accordance with the Japanese standard [7]) 3 h postoperatively and continued until postoperative day (POD) 6. (Fig. 2) Warfarin was also started when platelet count exceeded 10 × 10^4^/μl on POD 4 and continued for 3 months. The postoperative course was uneventful, and there were no signs of thromboembolism. PF4/H Ab was positive on POD 8 (examined by latex flocculation turbidimetry; 2.8 U/ml: the threshold is < 1.0) and negative by POD 91.

Informed consent was obtained from the patient for the reporting of this case.

## Discussion

Our patient’s clinical course suggested that in a ‘‘remote’’ HIT case, OPCAB could be carried out safely with the combination of perioperative argatroban and intraoperative heparin.

HIT is an antibody-mediated adverse drug reaction that is relatively common and has devastating comorbidity [2]. Platelet-activating immunoglobulin G (IgG) recognizes PF4 /H Ab complexes and causes HIT [2]. One of the characteristics of PF4/H Ab is its transient nature (median time to negative is 85 days) [3]. Therefore, surgery should be delayed for 3 months, unless it is urgent.

Although approximately 50% of patients who undergo cardiac surgery will eventually become HIT antibody-positive [1], only 1–2.4% will develop clinical HIT or HIT-T [1–4]. In general, in cardiac surgery, the platelet count decreases by approximately 38% immediately after CPB and continues to decrease for the first 1–2 PODs [1, 8]. If thrombocytopenia persists for 4 days postoperatively, HIT or HIT-T should be suspected [1].

In case of urgent cardiac surgery in a patient with HIT, ACCP guidelines suggest the use of bivalirudin over other non-heparin anticoagulants or heparin plus antiplatelet agents (Grade 2C) [1]. Furthermore, the EVOLUTION-On study reported no difference in the clinical outcome of heparin and bivalirudin in CPB [9]. Unfortunately, bivalirudin is not available in Japan; therefore, argatroban is the first choice.

In cases with a history of HIT with absent heparin antibodies, the management strategies differ. After testing negative for PF4/H Ab, the status is termed as ‘remote’ HIT and only short-term use of heparin is suggested over non-heparin anticoagulants (Grade 2C) [1]. Some studies have reported re-exposure to heparin in ‘remote’ HIT cases but the recurrence of HIT in cardiac/vascular surgery cases seems to be relatively low compared to the general incidence (1–2.4%) [1–4], with an incidence of 0–5% for HIT and 0% for HIT-T [2, 10–12]. Although PF4/H Ab was detected on POD 8 in our case (Fig. 1), appropriate postoperative anticoagulation prevented HIT-T. Since long-term use of heparin is a risk factor for HIT [1], the use of heparin should be limited to a very short period intraoperatively.

In the present case, heparin was used only during the intraoperative phase, and argatroban was considered as a perioperative anticoagulation. The course of ACT with heparin was normal (Fig. 1). The ACT data must be carefully taken in combination with actual observation of the operative field, always checking for sufficient anticoagulation.

Argatroban is metabolized by the liver [13], without neutralization. There is no standard control protocol for the use of argatroban, such as for heparin and bivalirudin [14]. Although argatroban has a short half-life (40–50 min) as compared to heparin [13], the anticoagulation effect remains longer than expected, and postoperative bleeding increases [15]. Moreover, the anticoagulation effect sometimes tends to be poor, and the risk of thrombotic occlusion of the CPB circuit increases [16]. Argatroban is not the preferred option in HIT cases, especially during the intraoperative phase, but is acceptable during the pre- and post-operative phases when other options are limited.

Postoperative anticoagulants are recommended for planned re-exposure to heparin for cardiac surgery with ‘remote’ HIT case [2], and when the Vitamin K antagonist is used, it should be initiated after platelets have substantially recovered [1]. Although taking warfarin until PF4/H Ab becomes negative increases the bleeding risk, we continued, because the antibody turned POD 8.

This patient was treated with OPCAB; however, in case of emergency conversion to on-pump CABG, peripheral (via the femoral artery and vein) extracorporeal membrane oxygenation (ECMO) should be considered, since clot formation in central (via the aorta and vena cava) ECMO will result in immediate hemodynamic collapse and increase the risk of cerebral infarction. Minimizing the dose of heparin by choosing peripheral ECMO is another way to decrease the risk of HIT.

## Conclusion

We present a case of a patient with a history of ‘remote’ HIT-T who underwent successful OPCAB with heparin and argatroban. Although this is a single case report, we consider this anticoagulation strategy to be effective especially in countries, where bivalirudin is not available. Re-exposure to heparin in cardiovascular surgery for patients with a history of ‘remote HIT’ is reasonable, and appropriate anticoagulation is important for an uneventful postoperative course.

## Data Availability

Not applicable.

## Abbreviations

ACT: Activated clotting time,
ACCP: American college of chest physicians
CPB: Cardiopulmonary bypass
CABG: Coronary artery bypass grafting
ECMO: Extracorporeal membrane oxygenation
HIT: Heparin-induced thrombocytopenia
HIT-T: Heparin-induced thrombocytopenia with thrombosis
MI: Myocardial infarction
OPCAB: Off-pump coronary artery bypass
PCI: Percutaneous coronary intervention
PF4/ H Ab: Platelet factor-4/heparin antibodies
POD: Postoperative day
UAP: Unstable angina pectoris

## References

[1] Linkins LA, Dans AL, Moores LK, Bona R, Davidson BL, Schulman S (2012). Treatment and prevention of heparin-induced thrombocytopenia: Antithrombotic Therapy and Prevention of Thrombosis, 9th ed: American College of Chest Physicians Evidence-Based Clinical Practice Guidelines. Chest.

[2] Warkentin TE, Anderson JA (2016). How I treat patients with a history of heparin-induced thrombocytopenia. Blood.

[3] Warkentin TE, Kelton JG (2001). Temporal aspects of heparin-induced thrombocytopenia. N Engl J Med.

[4] Warkentin TE, Kelton JG (1996). A 14-year study of heparin- induced thrombocytopenia. Am J Med.

[5] Wallis DE, Workman DL, Lewis BE, Steen L, Pifarre R, Moran JF (1999). Failure of early heparin cessation as treatment for heparin-induced thrombocytopenia. Am J Med.

[6] Cuker A, Arepally GM, Chong BH, Cines DB, Greinacher A, Gruel Y (2018). American Society of Hematology 2018 guidelines for management of venous thromboembolism: heparin-induced thrombocytopenia. Blood Adv.

[7] Pharmaceuticals and Medical Devices Agency. Japanese Pharmacopoeia 18th Edition. 2021. https://www.pmda.go.jp/english/rs-sb-std/standards-development/jp/0029.html.

[8] Nader ND, Khadra WZ, Reich NT, Bacon DR, Salerno TA, Panos AL (1999). Blood product use in cardiac revascularization: comparison of on- and off-pump techniques. Ann Thorac Surg.

[9] Dyke CM, Smedira NG, Koster A, Aronson S, McCarthy HL, Kirshner R (2006). A comparison of bivalirudin to heparin with protamine reversal in patients undergoing cardiac surgery with cardiopulmonary bypass: the EVOLUTION-ON study. J Thorac Cardiovasc Surg.

[10] Pötzsch B, Klövekorn WP, Madlener K (2000). Use of heparin during cardiopulmonary bypass in patients with a history of heparin-induced thrombocytopenia. N Engl J Med.

[11] Dhakal P, Giri S, Pathak R, Bhatt VR (2015). Heparin reexposure in patients with a history of heparin-induced thrombocytopenia. Clin Appl Thromb Hemost.

[12] Nuttall GA, Oliver WC, Santrach PJ, McBane RD, Erpelding DB, Marver CL (2003). Patients with a history of type II heparin-induced thrombocytopenia with thrombosis requiring cardiac surgery with cardiopulmonary bypass: a prospective observational case series. Anesth Analg.

[13] Hursting MJ, Soffer J (2009). Reducing harm associated with anticoagulation: practical considerations of argatroban therapy in heparin-induced thrombocytopenia. Drug Saf.

[14] Koster A, Dyke CM, Aldea G, Smedira NG, McCarthy HL, Aronson S (2007). Bivalirudin during cardiopulmonary bypass in patients with previous or acute heparin-induced thrombocytopenia and heparin antibodies: results of the CHOOSE-ON trial. Ann Thorac Surg.

[15] Matsuyama K, Kuinose M, Maruno K, Takahashi S, Toguchi K, Iwahashi T (2013). Difficulty in the management of anticoagulation with argatroban during off-pump coronary artery bypass grafting. J Cardiol Cases.

[16] Follis F, Filippone G, Montalbano G, Floriano M, Lobianco E, D'Ancona G (2010). Argatroban as a substitute of heparin during cardiopulmonary bypass: a safe alternative?. Interact Cardiovasc Thorac Surg.

